# Circumvent the uncertainty in the applications of transcriptional signatures to tumor tissues sampled from different tumor sites

**DOI:** 10.18632/oncotarget.15754

**Published:** 2017-02-27

**Authors:** Jun Cheng, You Guo, Qiao Gao, Hongdong Li, Haidan Yan, Mengyao Li, Hao Cai, Weicheng Zheng, Xiangyu Li, Weizhong Jiang, Zheng Guo

**Affiliations:** ^1^ Department of Bioinformatics, Key Laboratory of Ministry of Education for Gastrointestinal Cancer, Fujian Medical University, Fuzhou, 350001, China; ^2^ Department of Preventive Medicine, School of Basic Medicine Sciences, Gannan Medical University, Ganzhou, 341000, China; ^3^ Department of Colorectal Surgery, Fujian Medical University Union Hospital, Fuzhou, 350001, China

**Keywords:** the proportions of tumor epithelial cell, gene pairs, REOs-based signature, stromal cells, microdissected samples

## Abstract

The expression measurements of thousands of genes are correlated with the proportions of tumor epithelial cell (PTEC) in clinical samples. Thus, for a tumor diagnostic or prognostic signature based on a summarization of expression levels of the signature genes, the risk score for a patient may dependent on the tumor tissues sampled from different tumor sites with diverse PTEC for the same patient. Here, we proposed that the within-samples relative expression orderings (REOs) based gene pairs signatures should be insensitive to PTEC variations. Firstly, by analysis of paired tumor epithelial cell and stromal cell microdissected samples from 27 cancer patients, we showed that above 80% of gene pairs had consistent REOs between the two cells, indicating these REOs would be independent of PTEC in cancer tissues. Then, by simulating tumor tissues with different PTEC using each of the 27 paired samples, we showed that about 90% REOs of gene pairs in tumor epithelial cells were maintained in tumor samples even when PTEC decreased to 30%. Especially, the REOs of gene pairs with larger expression differences in tumor epithelial cells tend to be more robust against PTEC variations. Finally, as a case study, we developed a gene pair signature which could robustly distinguish colorectal cancer tissues with various PTEC from normal tissues. We concluded that the REOs-based signatures were robust against PTEC variations.

## INTRODUCTION

Based on gene expression profiles of clinical tumor tissues, many transcriptional signatures for cancer diagnoses [1] and prognoses [2–5] have been identified. Currently, most of transcriptional signatures are based on risk scores summarized from expression levels of the signature genes measured in clinical tumor tissues which are composed of tumor epithelial cells and stromal cells. However, because the proportions of tumor epithelial cell (PTEC) within different tumor sites are very different for the same tumor tissue [6] and epithelial cells and stromal cells in tumor tissues have different gene expression patterns [7, 8], the risk score for a patient could vary greatly in tumor tissues sampled from different tumor sites and thus tend to make unreliable risk prediction.

Several approaches have been proposed to reduce the influence of the PTEC in tumor tissues, including sampling with quality control, using the laser capture microdissection (LCM) technology to isolate pure tumor epithelial cells and using the deconvolution algorithm to estimate gene expression profiles of tumor epithelial cells from bulk tumor tissues. However, all these approaches have critical limitations. For sampling with quality control, the requirement of at least 60% [9] or 70% [10, 11] of tumor epithelial cells in tumor samples is quite rough and the common evaluation method of hematoxylin-eosin staining (HE) is rather subjective and uncertain [12, 13]. In addition, for highly heterogeneous tumors, such as pancreatic adenocarcinoma and diffuse gastric cancer, it is hard to meet the sample quality criteria. The LCM technology can acquire a few pure tumor epithelial cells [14], but the LCM is expensive and time consuming, which makes it difficulty to be widely used in clinical settings. Although several deconvolution algorithms have been proposed to decompose gene expression profiles into cell-type specific profiles [15, 16], their applications are limited because the absolute expression measurements of cell-specific signature genes are sensitive to experimental batch effects [17].

In general, experimental batch effects make a major barrier to the application of the type of prognostic signatures based on risk scores summarized from the expression level measurements of signature genes. Especially, current data normalization algorithms for adjusting batch effects will introduce substantial uncertainty for the prognostic prediction of patients [18] and the classification of patients into known disease subtypes [19], depending on risk or subtype composition of the samples adopted for normalization together. In contrast, another type of prognostic signatures is based on the relative expression orderings (REOs) of gene pairs within individual samples, which is robust against experimental batch effects and invariant to monotonic data normalization [20–22]. Based on REOs, some methods such as TSP [21], k-TSP [22] and other adjusted methods [19, 23] have been proposed to identify disease signatures, usually based on two categories of patients [24–27]. Recently, avoiding the subjective pre-grouping of samples into high- and low-risk groups, we have employed various tuned or adjusted strategies to identify REOs-based prognostic signatures for specific medical problems of different cancers. The identified REOs-based prognostic signatures for colorectal cancer [28], non-small cell lung cancer [18], ER+ breast cancer [29] and other cancers [30, 31] have been successfully verified in multiple normalization-free data produced by different laboratories, providing strong evidences of the clinical feasibility of this type of prognostic signatures.

In this article, considering that tumor stromal cells and tumor epithelial cells show a mass of similar differential REOs of gene pairs in comparison with their normal controls [32], we supposed that the REOs of gene pairs within individual samples could also be robust against PTEC variations given that the variations are not too large. Using data for colon cancer and breast cancer, we firstly demonstrated that the expression measurements of thousands of genes were significantly correlated with PTEC. Then, we showed that above 80% of all gene pairs had consistent REOs between every paired samples of tumor epithelial and stromal cells microdissected from cancer tissues and thus these REOs would be independent of PTEC in cancer tissues. Afterward, we did simulation experiments to confirm that much more REOs of gene pairs, especially those with larger expression differences in tumor epithelial cells, would remain unchanged in the tumor samples even when the PTEC decreased to as low as 30%. Finally, as a case study, we identified a REOs-based signature which could robustly distinguish colorectal cancer samples with various PTEC from normal tissues. Thus, the REOs-based signatures could be robustly converted to clinical application.

## RESULTS

### Gene expression measurements widely correlated with PTEC

Using 278 colon cancer samples with PTEC data from TCGA, we found the expression measurements of 2271 genes (see Supplementary Table 2) were significantly correlated with PTEC (Spearman correlation analysis, FDR<10%). Similarly, using 1076 TCGA samples with PTEC data for breast cancer, we found 2309 genes (see Supplementary Table 2) that their expression measurements were significantly correlated with PTEC (Spearman correlation analysis, FDR<10%). Notably, 840 of the genes correlated with PTEC in the colon tumor samples were also correlated with PTEC in the breast cancer samples, of which 98.81% had the same positive or negative correlation coefficients with PTEC in the two types of cancer tissues. The KEGG functional pathway enrichment analysis showed that these overlapped genes were significantly enriched in 13 pathways, including “Tyrosine metabolism”, “Focal adhesion” and some signaling ways (Supplementary Table 3), which could reflect the functional difference between tumor epithelial cells and tumor stromal cells [8, 32].

The correlations of gene expression measurements with PTEC could be introduced by genes differentially expressed between tumor epithelial cells and stromal cells. To validate this, we identified 6674 differentially expressed genes (DEGs) (Student's t-test, FDR<10%) between colorectal tumor epithelial cells and tumor stromal cells using the microdissected data from the GSE35602 dataset. Among these genes, the expression measurements of 1234 genes were also significantly correlated with PTEC (Spearman correlation analysis, FDR<10%). The concordance score of positive correlations with up-regulations and negative correlations with down-regulations was 98.62%, which was highly unlikely to be observed by chance (Binomial test, *P*-value<2.2×10^−16^, see Materials and Methods). We did the same analysis using the nine paired profiles of the tumor epithelial cell and stromal cell of invasive breast cancer from the GSE14548 dataset. The result showed that, among the 4288 DEGs between the tumor epithelial cells and stromal cells (Student's t-test, FDR<10%), the expression measurements of 567 genes were also correlated with PTEC (Spearman correlation analysis, FDR<10%) and the concordance score of positive correlations with up-regulations and negative correlations with down-regulations was 94.53% (Binomial test, *P*-value<2.2×10-16). Similarly, we also detected 8728 DEGs (Student's t-test, FDR<10%) between the 10 paired profiles of epithelial cells and tumor stromal cells for triple negative breast tumor from the GSE81838 dataset [33], among which 1159 genes were significantly correlated with PTEC and the concordance score was 93.53% (Binomial test, *P*-value<2.2×10-16). These results confirmed that the correlation of gene expression measurements with PTEC is mainly introduced by the difference between tumor epithelial cells and stromal cells.

### REOs of gene pairs are highly robust against PTEC variations

Then, using the GSE31279 dataset for eight paired expression profiles of the tumor epithelial cells and stromal cells microdissected from eight patients with colorectal cancer, we evaluated the consistency of REOs of gene pairs between the two types of cells extracted from each patients. The results showed that the concordance scores took values ranging from 82.02% to 89.40%. Similarly, for the nine paired invasive breast cancer samples from the GSE14548 dataset, the concordance scores of REOs between the two types of cells were 86.72-91.22% for all measured gene pairs. Also, for the 10 paired triple negative breast tumor samples from the GSE81838 dataset, the concordance scores of REOs between the two types of cells were 85.38%-93.24% for all measured gene pairs. Obviously, if the REOs of gene pairs in the tumor epithelial cells are consistent with those in the stromal cells, then they will not be affected by PTEC. Thus, above 80% of REOs of gene pairs in clinical tumor tissues would remain unchanged as PTEC varies.

**Figure 1:**
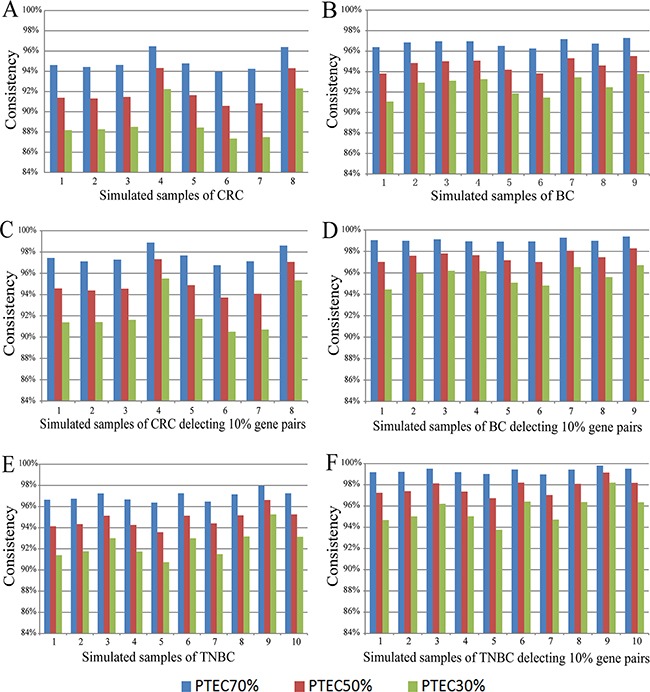
The consistency scores of REOs between tumor epithelial cells and simulated tumor tissue samples with 70%, 50% and 30% tumor epithelial cells The scores for all the gene pairs for the simulated colorectal tumor tissues **(A)**, for the simulated invasive breast tumor tissues **(B)** and for the simulated triple negative breast tumor tissues **(E)**. After deleting 10% of gene pairs that had the smallest expression rank differences in tumor epithelial cells, the increased scores for the remaining gene pairs for the simulated colorectal tumor tissues **(C)**, for the simulated invasive breast tumor tissues **(D)** and for the simulated triple negative breast tumor tissues **(F)**. The samples were simulated based on the paired samples of tumor epithelial cells and stromal cells from GSE31279, GSE14548 and GSE81838, and the description of the simulated samples corresponding to the paired samples is listed in Supplementary Table 1. CRC represents the colorectal tumor tissues, BC represents the invasive breast tumor tissues and TNBC represents the triple negative breast tumor tissues.

**Figure 2:**
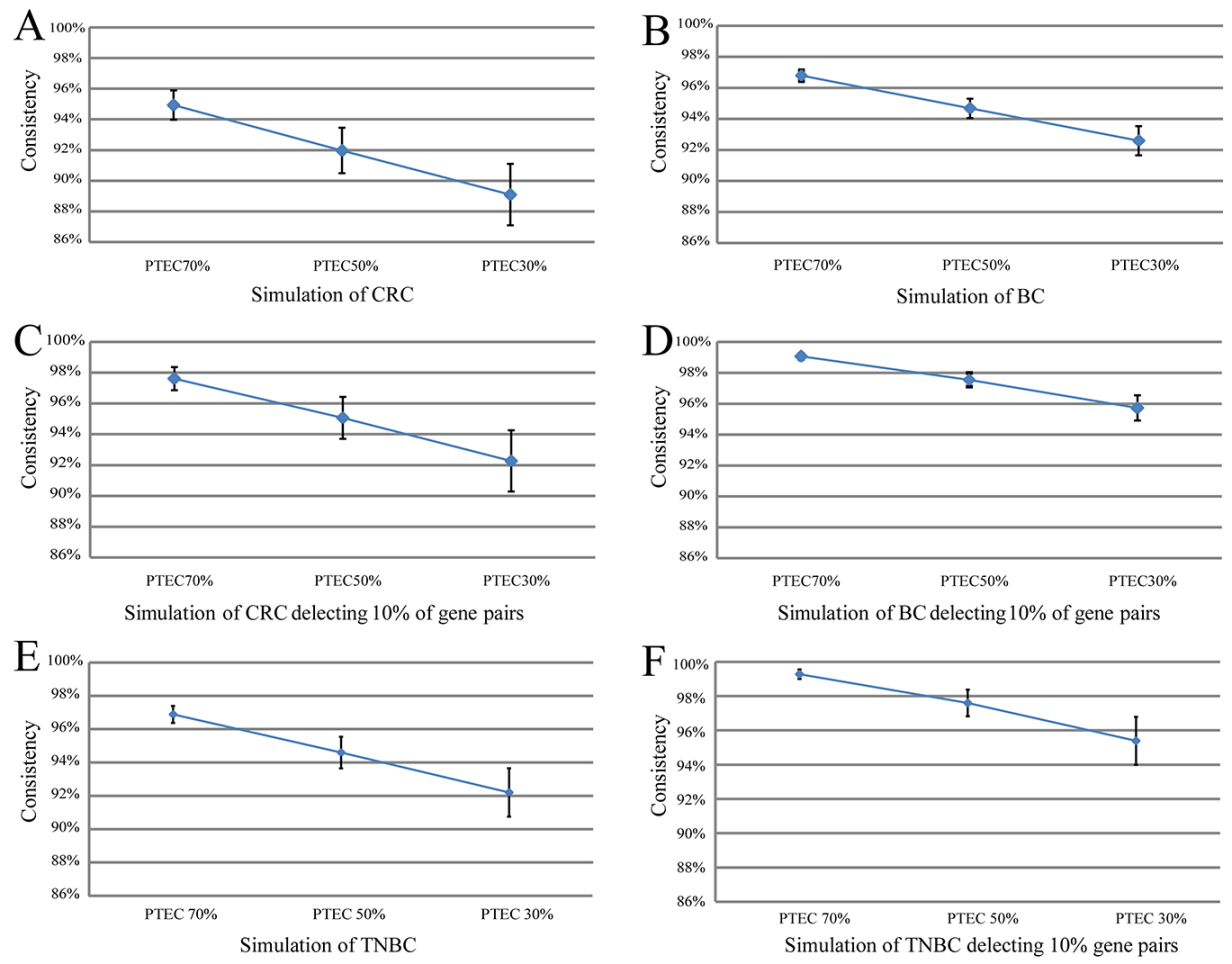
The summary line plot for means and SD of all the simulated sample consistency scores with 70%, 50% and 30% tumor epithelial cells The means and SD of all the simulated sample consistency scores with 70%, 50% and 30% tumor epithelial cells in Figure 2 **(A, B, E)** and after deleting 10% of gene pairs that had the smallest expression rank differences in tumor epithelial cells, the means and SD of all the simulated sample consistency scores with 70%, 50% and 30% tumor epithelial cells in Figure 2 **(C, D, F)**.

Then, we did simulation experiments to demonstrate that much more REOs of gene pairs, especially those with larger expression differences in the tumor epithelial cells, would not be readily disrupted by PTEC variations. Using each of the eight paired profiles of tumor epithelial and stromal cells for colorectal cancer, by successively replacing 10% of tumor epithelial cells with stromal cells, we generated gene expression profiles of simulated mixed-cell tumor tissues with decreased PTEC following equation (2) (see Material and Methods), and then evaluated the stability of REOs of gene pairs as PTEC decreased. As shown in Figure 1A, for the simulations based on the data for the eight patients, at least 93.96% of the REOs of gene pairs in the simulated tumor tissues with 70% of PTEC were consistent with their REOs in the corresponding tumor epithelial cells. When PTEC decreased to 50%, at least 90.57% of REOs of gene pairs in the corresponding tumor epithelial cells were unchanged in the eight individual simulations. Even when PTEC decreased to 30%, at least 87.34% of REOs of gene pairs were still maintained in the eight individual simulations. As shown in Figure 1C, after deleting 10% of gene pairs with the smallest absolute expression rank differences in each patient's tumor epithelial cell, at least 96.76%, 93.71% and 90.50% of REOs of gene pairs were stable when PTEC were 70%, 50% and 30% in the eight simulation experiments for colorectal tumor tissue. Similar simulation results were observed based on the GSE14548 dataset for nine paired profiles of the tumor epithelial cell and stromal cell extracted from invasive breast cancer patients, as shown in Figure 1B and Figure 1D. Similarly, based on each of the 10 paired tumor epithelial cells and stromal cells extracted from 10 triple negative breast cancer patients (the GSE81838 dataset), the simulation experiments showed that at least 96.37%, 93.57% and 90.74% of the REOs of gene pairs were unchanged when PTEC of the simulated tumor tissues were 70%, 50% and 30%, respectively. After deleting 10% of gene pairs with the smallest absolute expression rank differences in each patient's tumor epithelial cell, at least 99.02%, 96.74% and 93.75% of the REOs for all the measured gene pairs were unchanged when PTEC were 70%, 50% and 30%, as shown in Figure 1 and Figure 2.

**Figure 3:**
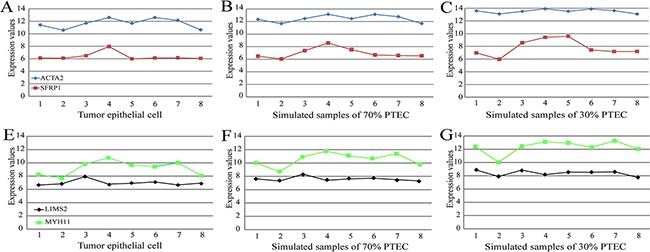
The influence of PTEC on the expression measurements and the REO of two pairs genes (*SFRP1* and *ACTA2*) and (*LIMS2* and *MYH11*) in colorectal samples **(A)** The expression measurements and the REO of *SFRP1* and *ACTA2* in pure tumor epithelial cells. **(B)** The expression measurements and the REO of *SFRP1* and *ACTA2* in simulated tumor tissues with 70% of PTEC. **(C)** The expression measurements and the REO of *SFRP1* and *ACTA2* in simulated tumor tissues with 30% of PTEC. The red nodes denote the gene of *SFRP1* and the blue nodes the gene of *ACTA2*. The simulated tissue samples with 70% or 30% PTEC were simulated based on eight paired samples of tumor epithelial cells and stromal cells from GSE31279. Similarly, the expression measurements and the REO of *LIMS2* and *MYH11* were showed in Figure 3 **(E-G)**. And the description of the tumor epithelial cells numbered 1-8 and simulated samples numbered 1-8 is listed in Supplementary Table 1.

Finally, in the simulated colorectal tumor data for the eight patients, we demonstrated the robustness of REOs against PTEC variations by analyzing two genes, *SFRP1* remarkably under-expressed in colorectal tumor tissues [34] and *ACTA2* over-expressed in malignant colorectal tumor stromal cells [35]. As shown in Figure 3, the expression measurements of the two genes were markedly affected by the PTEC, and the largest fold changes of gene expression levels of *SFRP1* and *ACTA2* in the simulated samples with 70% PTEC, compared with their expression levels in the corresponding tumor epithelial cells, were 1.26 and 1.10, respectively; while the largest fold changes dramatically increased to 1.60 and 1.24, when the PTEC in the simulated samples decreased to 30%. However, the REOs of these two genes within each of the eight simulated samples remained unchanged even when PTEC was 30%. In addition, we also analyzed a pair of genes, *LIMS2*, down-regulated in colon cancer epithelial cells compared with normal epithelial colon cells [36], and *MYH11* significantly up-regulated in colorectal tumor tissues compared with normal colon tissues [37]. As shown in Figure 3, compared with their expression levels in the corresponding tumor epithelial cells, the largest fold changes of gene expression levels of *LIMS2* and *MYH11* in the simulated samples with 70% PTEC were 1.14 and 1.22, respectively; while the largest fold changes increased to 1.34 and 1.51 when the PTEC in the simulated samples decreased to 30%. In contrast, the REOs of these two genes within all the eight simulated samples remained unchanged even when PTEC decreased to 30%, as illustrated in Figure 3.

### A case study for a REOs-based signature for colorectal cancer diagnosis

The above results showed that REOs of gene pairs are robust against PTEC variations, indicating that REOs-based tumor transcriptional signatures will be highly likely to be applicable when the sample quality is quite low. As a case study, we developed a gene pair signature to distinguish colorectal cancer samples from normal samples measured by different laboratories.

We used 177 tumor samples collected from the GSE17536 dataset and 84 normal samples collected from four datasets (GSE4183, GSE8671, GSE9254 and GSE21510) as the training data. Firstly, we selected 153,912,895 gene pairs each with the same REO in more than 95% of the tumor samples and 121,990,135 gene pairs each with the same REO in more than 95% of the normal samples. The two lists of gene pairs had 1,012,296 overlaps which showed reversal REOs between normal tissues and tumor tissues. From these gene pairs, we selected a gene pair (*CLDN8* and *MMP3*) with the largest reversal degree between the normal and tumor samples, defined as the signature (see Materials and Methods). If *CLDN8* has a higher expression measurement than *MMP3* in a sample, the sample is identified as a normal sample; otherwise, a tumor sample. In the training data, 98.31% of the tumor samples and 98.81% of the normal samples were correctly predicted by this signature.

**Figure 4:**
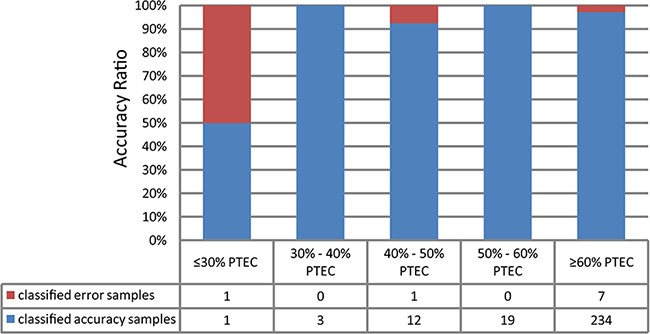
The validation rate binned by tumor cellularity for the tumor/normal signature validation on the 278 TCGA samples

We validated the signature using the 278 colon cancer samples with PTEC data and 41 normal samples from TCGA. The results showed that 96.76% of the tumor samples and 92.68% of the normal samples were correctly predicted. Notably, as showed in Figure 4, 35 of the 37 tumor samples with less than 60% of PTEC, including 15 samples with 30%-50% of PTEC, were correctly predicted. This indicated that the signature could predict correctly two types of samples with low PTEC. We further validated this signature using 628 colorectal tumor samples derived from GSE39582 and GSE35896 and 78 normal samples collected from two datasets (GSE56789 and GSE37364). The results showed that 96.02% of the 628 colorectal tumor samples and 97.44% of the 78 normal samples were correctly predicted. The details of the prediction accuracies for all the validation datasets were described in Table 1.

**Table 1:** The prediction accuracies for the validation data

Data set	Sample size		prediction accuracy
	Tumor	Normal	
TCGA	278	—	97.12%
TCGA	—	41	92.68%
GSE39582	566	—	95.76%
GSE35896	62	—	98.39%
GSE56789	—	40	100%
GSE37364	—	38	94.74%

## DISCUSSION

As demonstrated in this study, the expression measurements of thousands of genes are significantly correlated with PTEC in clinical tumor tissues. Thus, for transcriptional signatures based on risk scores summarized from expression levels of signature genes, the diagnostic or prognostic predictions for a patient could be uncertain when using tissues sampled from different sites of the patient's tumor. In contrast, we showed that about 90% of REOs of gene pairs in tumor epithelial cells can be maintained in clinical tumor tissues even when PTEC decreases to as low as 30%. Especially, the REOs of gene pairs with larger expression differences in tumor epithelial cells tend to be more robust against PTEC variations in clinical tumor tissues. As demonstrated in the case study for colorectal cancer diagnosis, the identified REOs-based signature can be robustly used for analyzing samples with different PTEC. Certainly, because a small percentage of REOs in tumor epithelial cells could be disrupted in clinical tumor tissues as PTEC decrease, it should be still necessary to sample tumor tissues with as high PTEC as possible to provide reliable diagnoses or prognoses.

The robustness of REOs-based signatures against PTEC variations in clinical tumor tissues makes this type of signatures being more feasible to clinical application. Arguably, some subtle quantitative information of gene expression could be lost when gene expression levels are translated into REOs. However, the subtle quantitative information of gene expressions measured by current high-throughput as well as low-throughput biotechnologies is in fact quite error-prone and uncertain due to the serious problem of experimental batch effects [17] and data normalizations [38, 39]. In contrast, the type of REOs–based signatures are insensitive to batch effects and data normalizations [21, 22] and thus could provide more accurate patient-specific information for clinical applications [40]. In addition, different from the traditional risk-score based signatures, the REOs–based signatures could perform robustly in low quality disease samples such like the samples with low PTEC, as demonstrated in this study, and the disease samples (including formalin-fixed paraffin-embedded samples) with certain RNA degradation [41]. Taking these practical factors into consideration, we believe that it should be definitely necessary to develop the REOs-based signatures for all important cancer types, aiming at ultimately developing clinically feasible signatures for personalized cancer diagnoses or prognoses.

## MATERIALS AND METHODS

### Data preprocessing

All datasets analyzed in this research were downloaded from the Gene Expression Omnibus (GEO, http://www.ncbi.nlm.nih.gov/geo/) and The Cancer Genome Atlas (TCGA, http://cancergenome.nih.gov/), as described in detail in Table 2. The MBatch expression profiles with batch effects adjustment for two cancers types (colon and breast cancer) were download from the TCGA MBatch Web Portal (http://bioinformatics.mdanderson.org/tcgambatch/) [42] and the clinical information for each sample was downloaded from the TCGA data portal, they were used to detected the genes significantly correlated with PTEC. For the same solid tumor tissue sample (01A) with multiple slide information of ‘percentage of tumor cells’, we used the mean [43] of percentage of tumor cells within multiple slides in performing Spearman correlation analysis and if the information of ‘percentage of tumor cells’ in one of multiple slides was unavailable, the samples would be deleted.

**Table 2:** The datasets analyzed in the study

Data type	Accession	Platform	Sample size	
			Tumor	Normal
COAD^†^ (Mbatch^¶^)	TCGA	RNAseqV2	278	—
BRCA† (Mbatch^¶^)	TCGA	RNAseqV2	1076	—
CRC*	GSE39582	Affymetrix GPL570	566	—
CRC*	GSE17536	Affymetrix GPL570	177	—
CRC*	GSE35896	Affymetrix GPL570	62	—
CRC*	GSE8671	Affymetrix GPL570	—	32
CRC*	GSE9254	Affymetrix GPL570	—	19
CRC*	GSE21510	Affymetrix GPL570	—	25
CRC*	GSE56789	Illumina GPL10558	—	40
CRC*	GSE37364	Affymetrix GPL570	—	38
CRC*	GSE4183	Affymetrix GPL570	—	8
COAD^†^	TCGA	IlluminaHiseq_RNAseqV2	278	41
			**Tumor epithelial cell**	**Tumor stromal cell**
TNBC(LCM^§^)	GSE81838	Affymetrix GPL6244	10	10
BC(LCM^§^)	GSE14548	Affymetrix GPL1352	9	9
CRC*(LCM^§^)	GSE35602	Agilent GPL6480	13	13
CRC*(LCM^§^)	GSE31279	Illumina GPL6104	8	8

Note: ^¶^Mbatch represents the batch effect-free expression profiles; ^§^LCM represents the tumor tissues are microdissected by LCM technology. ^†^COAD and BRCA represent colon adenocarcinoma samples and breast invasive carcinoma from TCGA. *CRC represents the colorectal tumor tissues, BC represents the invasive breast tumor tissues, TNBC represents the triple negative breast tumor tissues.

The eight paired colorectal tumor epithelial cells and stromal cells microdissected samples from the GSE31279 dataset and the nine paired tumor epithelial cells and stromal cells of invasive breast cancer microdissected samples from the GSE14548 dataset were used to simulate the clinical tumor tissues with different PTEC, respectively. The other datasets except GSE35602 were used to train and validate the signature. The raw data (.CEL files) from each dataset measured by the Affymetrix platform was processed using the Robust Multi-array Average (RMA) algorithm [44]. For the data measured by the Illumina and Agilent platforms, the processed expression profiles were directly downloaded. Every Probe-set ID was mapped to an Entrez gene ID with the corresponding platform files. If multiple probe-sets were mapped to the same gene, the mean of multiple probe-sets was the expression value for the gene. Probe-set IDs with no corresponding to Entrez gene ID or Probe-set IDs that corresponding to more than one Entrez gene ID were deleted. And the other mRNA-seq profiles of level 3 measured by RNA-sequencing platform were downloaded from the TCGA web portal.

### Correlation analysis and differential expression analysis

The Spearman correlation analysis was used to assess the correlation between genes expression measurements and PTEC. The Student's *t*-test was used to detect DEGs between tumor epithelial cells and stromal cells. In the article, all *P* values were adjusted using Benjamini-Hochberg (BH) procedure [45] and the false discovery rate (FDR) was less than 10%.

### Concordance score

If the expression level of a gene was positively (or negatively) correlated with PTEC and correspondingly up-regulated (or down-regulated) in tumor epithelial cells versus tumor stromal cells, then we defined that the two observations were concordant, indicating that the observed correlation could be explained by the differential gene expression between the two types of cells. If *k* genes were both correlated with PTEC and differentially expressed between tumor epithelial cells versus tumor stromal cells, among which *s* genes had concordant observations, then the concordance score was calculated as *s/k*. The probability of observing the concordance score by chance was calculated by the cumulative binomial distribution model as following:

$$P=1-\sum_{i=0}^{s-1}{}_{i}^{k}{\left(Pe\right)}^{i}{\left(1-Pe\right)}^{k-i}$$ (1)

where *Pe*=0.5 is the probability of one gene having concordant observation in two types of gene lists by chance.

### Simulation of tumor samples with different PTEC

Based on each paired profiles of tumor epithelial cells profiles and stromal cells from the same patient, we produced data to simulate the clinical tumor tissues with different PTEC by the equation as following:

$$E=EePi+Es\left(1-Pi\right),\;Pi\hat{\text{ I}}\;(0,1)\;\left(2\right)$$

where *E, Ee* and *Es* represent the gene expression profiles of the simulated clinical tumor tissue, tumor epithelial cell and stromal cell, respectively. *Pi* is the PTEC in the simulated clinical tumor sample.

### Developing an REOs-based signature for distinguishing tumor samples from normal samples

Firstly, a gene pair (*Gi* and *Gj*) was selected when its REO, *Gi* > *Gj* in expression level, was identical in more than 95% of the normal samples, and was reversed (*Gi* <*Gj*) in more than 95% of the tumor samples. After selecting all such reversal gene pairs, we calculated for each gene pair the rank difference in each of the tumor or normal samples by the equations as following:

$$R_{ij}=\left|R_{i}-R_{j}\right|\;\left(3\right)$$

*R_i_* and *R_j_* represent the ranks of gene *i* and *j* in a sample, respectively, and *R_ij_* is the absolute rank difference between the two genes. Here, all the genes in a sample are ranked according to their expression levels in ascending order.

$$avgRij=\frac{median[Rij\left(N\right)]+median[Rij\left(T\right)]}{2}\;\left(4\right)$$

Let median [*Rij*(*N*)] and median [*Rij*(*T*)] represent the medians of absolute rank differences of the gene pair (*i*, *j*) in all normal samples and all tumor samples, respectively. Then, we calculated the arithmetic mean of the median [*Rij*(*N*)] and the median [*Rij*(*T*)] to evaluate the reversal degree of the gene pair. The larger this arithmetic mean, the larger the reversal degree of the REO for the two genes between the disease and normal tissues.

Finally, among all the reversal gene pairs, the gene pair with the largest arithmetic mean of the absolute rank differences in normal and cancer samples was selected as the signature. For a given sample, if the REO of the signature gene pair in the sample is the same with the REO pattern of the normal training samples, the samples was identified as the normal tissue; otherwise, the tumor sample.

## SUPPLEMENTARY TABLES





## Abbreviations

PTEC: proportions of tumor epithelial cell
REOs: relative expression orderings
LCM: laser capture microdissection
FDR: false discovery rate
DEGs: differentially expressed genes
FFPE: formalin-fixed paraffin-embedded
