# Supplementary material for: Circumvent the uncertainty in the applications of transcriptional signatures to tumor tissues sampled from different tumor sites
Source: Oncotarget. 2017 Feb 27;8(18):30265–75. doi: 10.18632/oncotarget.15754 (PMC5444741; doi:10.18632/oncotarget.15754)
Supplement: Supplementary file 1 [file oncotarget-08-30265-s001.pdf]

Circumvent the uncertainty in the applications of transcriptional signatures to tumor tissues sampled from different tumor sites

SUPPLEMENTARY TABLES

Supplementary Table 1: Paired samples of tumor epithelial cells and stromal cells for simulation

CRC (GSE31279)		BC (GSE14548)		TNBC (GSE81838)	
simulated sample1	GSM775184<br>GSM775185	simulated sample1	GSM363914<br>GSM363917	simulated sample1	GSM2176482<br>GSM2176492
simulated sample2	GSM775190<br>GSM775191	simulated sample2	GSM363920<br>GSM363923	simulated sample2	GSM2176483<br>GSM2176493
simulated sample3	GSM775224<br>GSM775225	simulated sample3	GSM363933<br>GSM363935	simulated sample3	GSM2176484<br>GSM2176494
simulated sample4	GSM775249<br>GSM775250	simulated sample4	GSM363937<br>GSM363939	simulated sample4	GSM2176485<br>GSM2176495
simulated sample5	GSM775258<br>GSM775259	simulated sample5	GSM363941<br>GSM363943	simulated sample5	GSM2176486<br>GSM2176496
simulated sample6	GSM775264<br>GSM775265	simulated sample6	GSM363958<br>GSM363961	simulated sample6	GSM2176487<br>GSM2176498
simulated sample7	GSM775270<br>GSM775271	simulated sample7	GSM363963<br>GSM363966	simulated sample7	GSM2176488<br>GSM2176497
simulated sample8	GSM775286<br>GSM775287	simulated sample8	GSM363968<br>GSM363971	simulated sample8	GSM2176489<br>GSM2176499
		simulated sample9	GSM363974<br>GSM363977	simulated sample9	GSM2176490<br>GSM2176500
				simulated sample10	GSM2176491<br>GSM2176501

Note: CRC represents colorectal tumor; BC represents invasive breast tumor; TNBC represents triple negative breast cancer.

Supplementary Table 2:The genes correlated with PTEC

See Supplementary File 1

Supplementary Table 3: The KEGG functional pathway of the 840 overlapped genes

Pathway	P
Tyrosine metabolism	0.0017924
Rap1 signaling pathway	0.0003001
Calcium signaling pathway	2.16E-06
cGMP-PKG signaling pathway	0.0006404
Neuroactive ligand-receptor interaction	1.90E-05
PI3K-Akt signaling pathway	3.85E-08
Adrenergic signaling in cardiomyocytes	0.000155
Vascular smooth muscle contraction	0.0003779
Focal adhesion	2.15E-07
ECM-receptor interaction	3.50E-14
Cell adhesion molecules (CAMs)	0.0016456
Platelet activation	0.0023672
Protein digestion and absorption	2.00E-06
